# On Malignant Tumours of the Rectum

**Published:** 1894-08-18

**Authors:** Alexander Miles

**Affiliations:** Surgical Tutor Royal Infirmary, Edinburgh


### MEDICAL PROGRESS AND HOSPITAL CLINICS.

[*The Editor will be glad to receive offers of co-operation and contributions from members of the profession. All letters should be addressed to* The Editor, The Lodge, Porchester Square, London, W.]

### ON MALIGNANT TUMOURS OF RECTUM.

By Alexander Miles, M.D., F.R.C.S.Edin., Surgical Tutor Royal Infirmary, Edinburgh.

Although modern pathologists unite in deprecating the use of the word "cancer" in descriptive surgery, it seems still, in spite of its vagueness, to retain its place even in the most recent text-books. Its use in connection with malignant neoplasms of the rectum seems, perhaps, more completely justifiable than in any other part of the body, because, although a number of different varieties of such growths are described, it seems exceedingly difficult to lay down any hard and fast pathological rules by which they are to be differentiated. A generic name is necessary, and "cancer" is perhaps as convenient a one as can be got.

If we exclude sarcoma, which is a very rare condition in the rectum, all the other varieties of malignant tumour—scirrhus, encephaloid, colloid, &c.—seem to differ from one another rather in minor details than in essence, all consisting in an exaggerated growth and abnormal arrangement of epithelial cells and fibrous stroma, the relative proportion of which determines the class into which a particular tumour shall go. The epithelium is of the same type as that which forms the Lieberkuhnian follicles, and is arranged after the form of these glands, hence the name adenoid-carcinoma is fitly applied to the group.

Some authorities[1] decline to go further in subdividing these tumours, and deny that typical scirrhus or medullary, or even sarcomatous tumours can be distinguished amongst rectal growths. When the fibrous tissue greatly predominates in amount, as it rarely does, the tumour is called *scirrhus*; when the stroma is scarce, and the overgrowth of epithelium great, it is spoken of as *encephaloid* or *medullary* cancer. The term *colloid* refers to a particular form of degeneration which the epithelial cells undergo in certain rare cases.

In the early stages of the growth, only a small portion of mucous membrane shows the increased epithelial formation, at the same time as the submucous tissue becomes infiltrated with leucocytes. As time goes on, the Lieberkuhn's follicles increase enormously in size and numbers, the new growth breaks through the limiting membrane, and infiltrates the thickened sub-mucous areolar tissue, the muscular layer of the rectal wall, and even the surrounding organs. The branching processes of the tumour pass irregularly in every direction, so that the growth does not assume any definite shape or boundary. At the same time it projects into the lumen of the bowel, and may grow along the rectal wall.

It is possible to classify these neoplasms according to their disposition in the rectal wall. Mr. Ball[2] subdivides them into (1) the *tuberous*, which is soft, contains little stroma, and projects as a fungating mass from one wall into the lumen of the bowel; (2) the *laminar* firmer, slower in its growth, and extending laterally, infiltrating as it goes: this he considers the commonest variety; (3) the *annular* encircles the bowel, constricting it, and showing little tendency to spread vertically.

**Frequency.**—Statistics show that 3·5 per cent. of all cases of cancer are met with in the rectum; and of cancers affecting the intestinal tract, 80 per cent. occur in the lowest part of the gut.[2]

**Sex.**—The opinion, or rather the experience of different observers, varies on the question of sex in relation to malignant rectal tumours. Some[3] have met with it oftenest in the female sex; others[4] in the male. On the whole, the sexes seem to be about equally prone to suffer from it.[2]

**Age.**—It is true that malignant growths are occasionally met with in the young, even under the age of twenty, but they undoubtedly much more frequently attack those advanced in years. Mr. Allingham[4] considers that it is in middle life that they are most frequent, and that when the disease does occur in the aged its growth is slow, and its symptoms less aggrevated.

**Heredity.**—That cancer is hereditary is a tradition commonly accepted by the laity. Mr. Cripps has critically examined the evidence on the point, and concludes that it is a fallacy. There are certainly cases which appear to support an opposite view, but they are not sufficiently numerous to exclude their being mere coincidences, and the cases in which the same disease has occurred in uncles, cousins, and other collateral relatives of the patient have no bearing on the question of heredity. Professor Annandale[5] has for many years pointed out in his clinical lectures that the parents of patients suffering from epithelial tumours are exceptionally long lived, a fact which goes to support Mr. Cripps' view.

**Symptoms.**—In its onset cancer of the rectum is an exceedingly insidious disease. As Mr. Cripps puts it, the first sign of it is that the patient becomes aware that he has a rectum. There is itching, slight pain at night or after exertion, with perhaps blood and slime passing with the motions. So long as the tumour bulges towards the interior of the rectum pain is slight, but when it has infiltrated the walls, and come to press on the pelvic contents, notably the nerves of the sacral pexus, it is a marked feature, especially if any inflammatory condition be superadded. Any involvement of the anus, with its abundant nerve supply, also increases this symptom. Bleeding indicates that ulceration has begun, and is seldom entirely absent, although on the other hand it is rarely severe. The escape of a sanious, sero-purulent, and putrid discharge, the odour of which is said to be characteristic, gives rise to much irritation of the skin around the anus. Certainly, one of the most characteristic symptoms of rectal carcinoma is that of alternate constipation and diarrhœa, with tenesmus. Complete obstruction is not common when the disease is located in the lower reaches of the rectum, because the tumour sloughs and so clears the passage. In the higher parts obstruction is more frequent. A digital examination, which should be made after the rectum has been emptied by an enema, will reveal the presence of a hard indurated and irregular mass, the lower part of which often feels like the os uteri. It is usually situated within three inches of the anus, although it seldom begins close down to the opening. No attempt is to be made to get the finger beyond the stricture—a step fraught with great danger. Not unfrequently it is near the sigmoid flexure and beyond the reach of the finger, in which case there is less pain and less desire to go to stool, but more vomiting, and constipation amounting even to obstruction. A bimanual examination helps in diagnosis[5] to determine the height to which the tumour extends.

**Differential Diagnosis.**—The disease under consideration has to be differentiated from (1) non-malignant tumours, (2) gummata, (3) hæmorrhoids. Simple tumours are commonest in the young, are usually of long standing and slow progress, and are for the most part pedunculated. The lymphatic glands are not implicated as in cancer. Much difficulty is often experienced in distinguishing between gummata and malignant tumours. Close enquiry into the previous history, the mode of onset and progress of the disease, together with evidence regarding other specific manifestations and the therapeutic test by potassium iodide will aid. A microscopic examination of scrapings or of fragments passed may confirm a diagnosis, but is seldom sufficiently definite to establish one. The diagnosis of hæmorrhoids is comparatively easy.

**Complications.**—In its growth a rectal cancer may involve the bladder, the prostate, or the vagina, giving rise to much discomfort. Peri-rectal and ischio-rectal suppuration, with perineal sinuses are not infrequent. The glands of the abdomen, groin, and pelvis are often enlarged, press on veins, and cause œdema of one or both legs. Secondary implication of the liver is common. Cramps and pains in the legs indicate pressure on the sacral pexus, and very often a patient comes complaining of sciatica, which a careful examination shows to be a manifestation of an unsuspected rectal cancer.

**Prognosis.**—Speaking generally, cancer of the rectum is not among the most malignant forms of tumour. The average duration of life without operation is about two years. The hard tumours are less rapid in their growth than the soft ones. Death may take place (1) from exhaustion from pain and discharge and cachexia; (2) from perforation setting up peritonitis; (3) from hæmorrhage; or (4) from obstruction.[3]

**Treatment.**—Being a disease with no tendency to spontaneous cure, extirpation is the only treatment which holds out a hope of permanent benefit. Caustics are worse than useless, acting only as additional irritants. The symptoms may be palliated by keeping the patient in bed or in a recumbent position. Appropriate diet to keep up the strength, and to avoid constipation; and opium in the form of suppository or hypodermically, are strongly indicated. When tenesmus causes much discomfort the sphincter may be divided. The operative procedures belong to two great classes: (1) Colotomy, which is palliative; and (2) excision, which is more or less radical. The consideration of these, however, must be meanwhile deferred.

[1] Cripp's "Diseases of Rectum and Anus." 2nd Ed., 1890. [2] Ball. "The Rectum and Anus." [3] Erichsen. "Science and Art of Surgery." 8th Ed. [4] Allingham. "Diseases of Rectum and Anus." 5th Ed., 1888. [5] Edin. Hosp. Rep. Vol. 2, p. 460.

